# Addition of terlipressin to norepinephrine in septic shock and effect of renal perfusion: a pilot study

**DOI:** 10.1080/0886022X.2022.2095286

**Published:** 2022-07-20

**Authors:** Jinlong Wang, Mengjuan Shi, Lili Huang, Qing Li, Shanshan Meng, Jingyuan Xu, Ming Xue, Jianfeng Xie, Songqiao Liu, Yingzi Huang

**Affiliations:** Jiangsu Provincial Key Laboratory of Critical Care Medicine, Department of Critical Care Medicine, Zhongda Hospital, School of Medicine, Southeast University, Nanjing, China

**Keywords:** Sepsis, terlipressin, renal perfusion, acute kidney injury, renal contrast-enhanced ultrasound

## Abstract

**Purpose:**

Terlipressin improves renal function in patients with septic shock. However, the mechanism remains unclear. Here, we aimed to evaluate the effects of terlipressin on renal perfusion in patients with septic shock.

**Materials and Methods:**

This pilot study enrolled patients with septic shock in the intensive care unit of the tertiary hospital from September 2019 to May 2020. We randomly assigned patients to terlipressin and usual care groups using a 1:1 ratio. Terlipressin was intravenously pumped at a rate of 1.3 μg/kg/hour for 24 h. We monitored renal perfusion using renal contrast-enhanced ultrasound (CEUS). The primary outcome was peak sonographic signal intensity (a renal perfusion parameter monitored by CEUS) at 24 h after enrollment.

**Results:**

22 patients were enrolled in this study with 10 in the terlipressin group and 12 in the usual care group. The baseline characteristics of patients between the two groups were comparable. The peak sonographic signal intensity at 24 h after enrollment in the terlipressin group (60.5 ± 8.6 dB) was significantly higher than that in the usual care group (52.4 ± 7.0 dB; mean difference, 7.1 dB; 95% CI, 0.4–13.9; adjusted *p* = .04). Patients in the terlipressin group had a lower time to peak, heart rates, norepinephrine dose, and a higher stroke volume at 24 h after enrollment. No significant difference in the urine output within 24 h and incidence of acute kidney injury within 28 days was found between the two groups.

**Conclusions:**

Terlipressin improves renal perfusion, increases stroke volume, and decreases norepinephrine dose and heart rates in patients with septic shock.

## Introduction

Septic shock is characterized by high morbidity and mortality in intensive care medicine [1–3]. Acute kidney injury (AKI) is a severe complication of septic shock associated with a poor prognosis [4,5]. Deterioration of renal hypoperfusion increases the occurrence and development of septic shock-associated AKI. Currently, the methods used to improve renal perfusion mainly focus on maintaining the stability of the macrocirculation. However, improvements in macrocirculation are not always accompanied by improvements in microcirculation [6]. Improving renal perfusion at microcirculation levels may be beneficial to prevent the deterioration of AKI in patients with septic shock.

Terlipressin is a synthetic vasopressin analog and has a great affinity for V1 receptors that are distributed in vascular smooth muscle and cause vasoconstrictive effects. However, the V1 receptor is heterogeneously distributed in the renal microcirculation. The vasoconstrictive effect of terlipressin on renal vessels is mainly concentrated on the efferent arterioles, whereas the effect on the afferent arterioles is negligible [7–9]. In addition, vasopressin is often relatively deficient in patients with septic shock [10]. Therefore, terlipressin supplementation may represent a promising method to improve renal perfusion in patients with septic shock [7].

Renal contrast-enhanced ultrasound is a noninvasive and safe method for bedside monitoring of renal perfusion. It has been proposed to quantify renal cortical perfusion in a variety of settings, including acute kidney injury and chronic kidney disease. During the contrast-enhanced ultrasound, the contrast agent increases blood echogenicity and enhances ultrasound visibility of renal microcirculation [11]. A good correlation has been observed between renal perfusion measured by contrast-enhanced ultrasound and gold standard renal blood flow measurements [12]. This study aimed to observe the effects of terlipressin added to usual care vs. usual care on renal perfusion monitored using renal contrast-enhanced ultrasound in patients with septic shock.

## Material and methods

### Study design

This pilot study used a parallel randomized controlled trial method and aimed to investigate the effects of terlipressin added to usual care vs. usual care on renal perfusion in patients with septic shock. We randomly assigned enrolled patients to the terlipressin group (terlipressin added to usual care) and the usual care group using a 1:1 ratio. The local clinical research ethics committee approved this study (2019ZDSYLL196-P01). Informed consent forms were signed by relatives of the participants by law. We registered this study at clinicaltrial.gov (NCT04948372). We present the following article following the CONSORT reporting checklist.

### Participants

From 1 September 2019, to 31 May 2020, we screened patients with septic shock daily at 7 a.m. and 7 p.m. in the intensive care unit (ICU) of the Zhongda Hospital affiliated with Southeast University. The inclusion criteria were adult patients (age ≥18 years old) with septic shock whose norepinephrine dose was greater than or equal to 15 μg/min. Exclusion criteria included age older than 85 years; serum creatinine level greater than 177 μmol/L; acute myocardial ischemia (acute myocardial infarction during shock, based on history, electrocardiogram, and enzyme parameters); acute mesenteric artery ischemia (unexplained abdominal pain and computed tomography suggesting calcification of the mesenteric artery or suspected mesenteric artery embolism); pregnancy; expected death within 24 h and no sign of informed consent. Septic shock was defined as an acute increase in sequential organ failure assessment (SOFA) score ≥2 points due to infection with hypotension requiring vasopressors to maintain mean arterial pressure (MAP) ≥65 mmHg despite adequate volume resuscitation [13].

### Randomization

We randomly assigned the enrolled patients to the terlipressin group and the usual care group using a 1:1 ratio. We used a random number table from 1 to 100 to randomize patients. The patient who was randomized to an odd number was assigned to the terlipressin group, and an even number was assigned to the usual care group. We used the envelope method to conceal the random results. The random results were placed in envelopes in order. When a patient was enrolled, we opened the corresponding envelope, reviewed the randomized results, and began the allocated intervention.

### Intervention

Patients in the terlipressin group received a fixed dose of terlipressin added to usual care. We dissolved terlipressin (0.86 mg) in 43 mL of 5% glucose solution (terlipressin concentration 20 μg/mL). Terlipressin was intravenously pumped at a fixed dose of 1.3 μg/kg/hour for 24 h. We adjusted the norepinephrine dose to maintain a MAP greater than 65 mmHg. Then, clinicians set the target MAP based on the clinical characteristics of the patients, and the norepinephrine dose was adjusted based on the target. Terlipressin was discontinued if the systolic blood pressure was greater than 160 mmHg for 30 min. Patients in the usual care group were treated with standard care. Norepinephrine was used as the vasoactive drug to maintain MAP. Septic shock was treated according to the international guidelines for the management of sepsis and septic shock, such as infection source control, fluid resuscitation, and the use of antibiotics [14].

### Outcome

The primary outcome was peak sonographic signal intensity at 24 h after enrollment. Peak sonographic signal intensity is a renal perfusion parameter monitored by renal contrast-enhanced ultrasound. Secondary outcomes included other renal perfusion parameters (time to peak, regional blood flow, and mean transit time at 24 h after enrollment); renal resistance index monitored by renal Doppler ultrasound at 24 h after enrollment; urine output within 24 h; and AKI within 28 days. AKI was defined as a serum creatinine increase ≥50% within seven days or an increase ≥26.5 μmol/L within 48 h from baseline [15]. Adverse events (arrhythmology, digital ischemia, diarrhea, and hyponatremia) during the ICU stay were also observed.

### Renal contrast-enhanced ultrasound

We monitored renal perfusion at the bedside using renal contrast-enhanced ultrasound. The ultrasound physicians performed renal contrast-enhanced ultrasound using Esaote MyLab Twice ultrasound with CA541 convex probe and SonoVue (Bracco, Italy) as the contrast agent. The ultrasound physicians were blinded to the grouping scheme. Renal perfusion was monitored at baseline and 24 h after enrollment by the same ultrasound physician. The interval between the two measurements in bilateral renal was at least 15 min. The detailed method of renal contrast-enhanced ultrasound is shown in Supplemental Appendix 1.

After the images were collected (Supplemental video 1), we imported the images into the QONTRAST software (Esaote, Italy) for analysis [16]. Three regions of interest in the renal cortex were collected for each image and the regions of interest were 25 square millimeters in size. Movement compensation was applied automatically. A time-intensity curve was generated and renal perfusion parameters were calculated based on this curve (Supplemental Figure 1).

### Statistical analysis

Continuous variables were expressed as the mean ± standard deviation or median (interquartile range) based on the data distribution. Categorical variables are expressed as numbers and percentages. The method of sample size calculation is shown in Supplemental Appendix 2. We used linear regression and binary logistic regression models to compare the differences in primary and secondary outcomes between the terlipressin and usual care groups. The differences in renal perfusion parameters at 24 h after enrollment were adjusted for baseline value, age, and acute physiology and chronic health evaluation II (APACHE II) score. Urine output within 24 h and AKI within 28 days were adjusted for age and APACHE II score.

We compared two sets of data using the independent or paired samples *t*-test, the Mann-Whitney U test, or Fisher’s exact test based on data type and distribution. For the repeated measurement data (number ≥ 3), we used ANOVA with two-way repeated measurements for data analysis. Kaplan-Meier curves were used to compare the occurrence of AKI within 28 days with the log-rank test between the terlipressin and usual care groups. A two-sided *p*-value < .05 was considered statistically significant. Statistical analysis was performed with SPSS (Version 23.0; IBM Corp.) and R (Version 3.6.3; R Project for Statistical Computing).

## Results

### Participants

**Figure 1.**
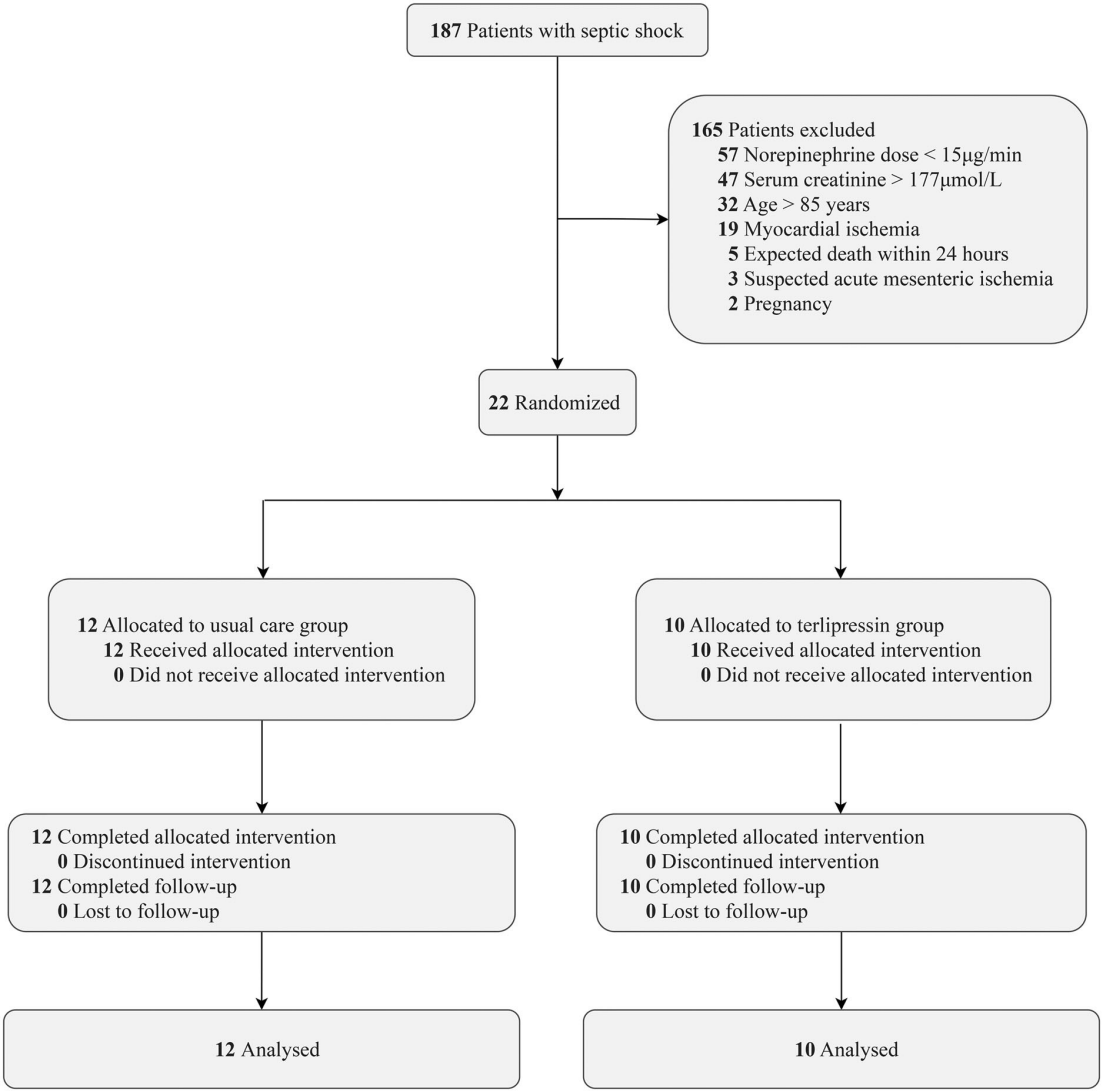
Flow diagram.

We screened 187 patients with septic shock. After excluding 165 patients, we randomly assigned 22 patients to the terlipressin group (*n* = 10) and the usual care group (*n* = 12). All randomized patients completed the allocated intervention and were included in the final analysis (Figure 1).

The baseline characteristics of patients between the terlipressin and usual care groups were comparable. No statistically significant differences in age, sex, height, weight, APACHE II score, SOFA score, infection sites, or previous coexisting diseases at baseline were noted between the two groups. No statistically significant differences were found in renal perfusion parameters at baseline between the two groups. No significant difference in 28-day mortality was noted between the two groups (Table 1).

**Table 1.** Characteristics of the patients, and renal perfusion at baseline.

Variables	Overall (*N* = 22)	Terlipressin (*N* = 10)	Usual care (*N* = 12)
Male, no. (%)	15 (68.2)	7 (70.0)	8 (66.7)
Age, years, mean ± SD	66.3 ± 15.2	61.7 ± 16.2	66.3 ± 15.2
Height, cm, mean ± SD	168.6 ± 7.1	169.2 ± 7.4	168.1 ± 7.2
Weight, kg, mean ± SD	67.3 ± 15	63.9 ± 15.8	70.1 ± 14.3
APACHE II score, mean ± SD	21 ± 6.8	18.6 ± 6.7	23.1 ± 6.4
SOFA score, mean ± SD	8.4 ± 2.3	8.8 ± 2.9	8 ± 1.7
Sites of infection, no. (%)			
Respiratory system	10 (45.5)	2 (20)	8 (66.7)
Abdominal	10 (45.5)	6 (60)	4 (33.3)
Urinary	1 (4.5)	1 (10)	0 (0)
Skin and soft tissue	1 (4.5)	1 (10)	0 (0)
Previous coexisting disease, no. (%)			
Hypertension	11 (50)	3 (30)	8 (66.7)
Diabetes	5 (22.7)	2 (20)	3 (25)
Coronary heart disease	5 (22.7)	1 (10)	4 (33.3)
Cerebrovascular disease	6 (27.3)	2 (20)	4 (33.3)
Renal perfusion^a^			
PI, dB, mean ± SD	56.5 ± 9.4	59.2 ± 7.0	54.1 ± 10.8
TTP, s, mean ± SD	19.3 (14.1, 22.8)	19.3 (15.0, 23.2)	19.5 (14.8, 21.8)
RBF, au, mean ± SD	75.7 ± 13.0	80.0 ± 9.5	72.1 ± 14.7
MTT, s, mean ± SD	55.4 (47.3, 78.0)	52.5 (47.4, 80.6)	64.2 (47.1, 76.7)
RRI, mean ± SD	0.70 ± 0.06	0.68 ± 0.05	0.71 ± 0.06
28-day mortality, no. (%)	9 (40.9)	4 (40)	5 (41.7)

^a^ PI, TTP, RBF, and MTT were monitored by renal contrast-enhanced ultrasound. RRI was monitored by renal Doppler ultrasound. APACHE II: acute physiology and chronic health evaluation II; MTT: mean transit time; PI: peak sonographic signal intensity; RBF: regional blood flow; RRI: renal resistance index; SD: standard deviation; SOFA: Sequential Organ Failure Assessment; TTP: time to peak.

### Primary outcome

**Figure 2.**
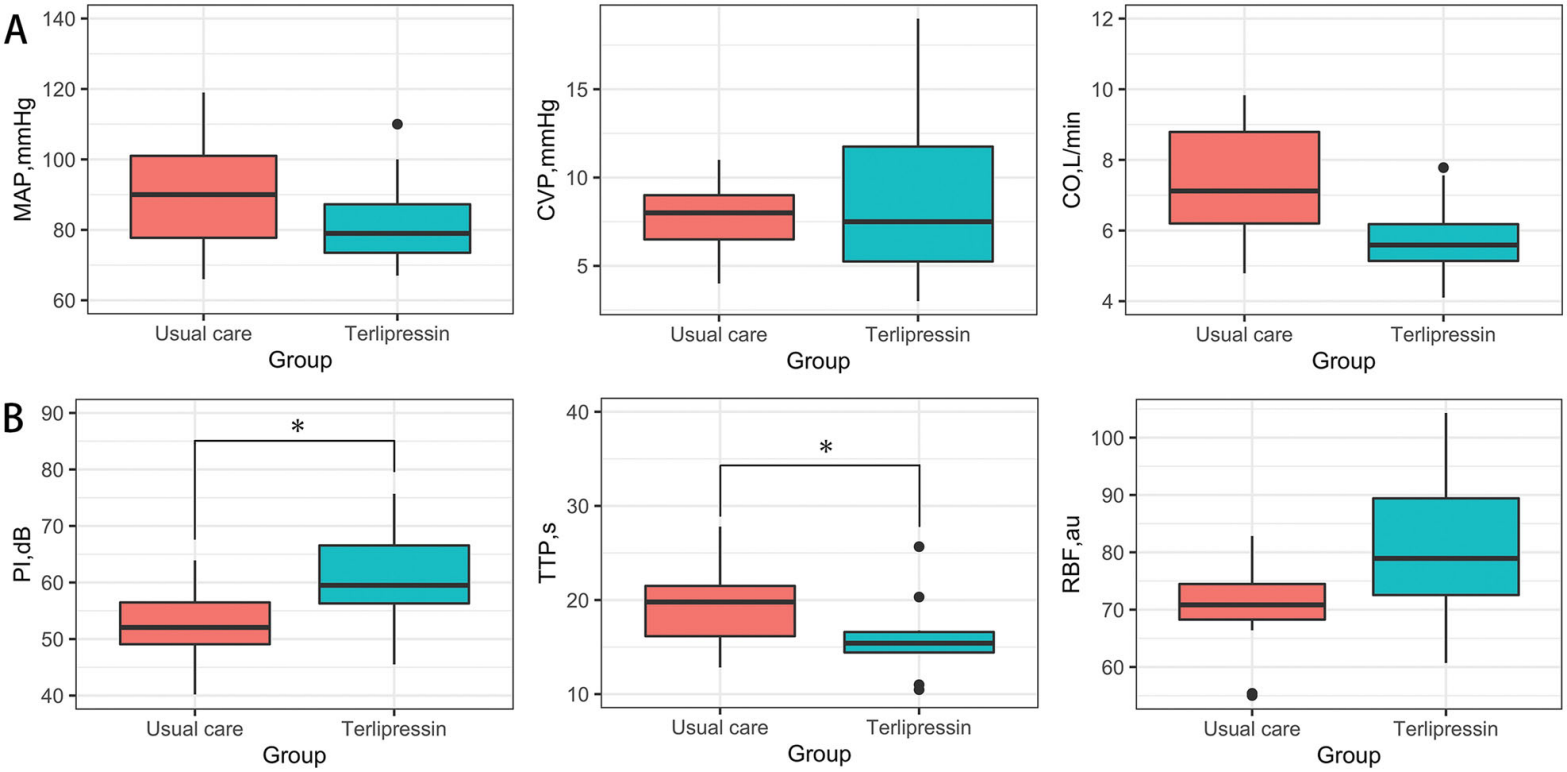
Effects of terlipressin on macrocirculation (A) and renal perfusion (B) at 24 h after enrollment in patients with septic shock. Macrocirculation was monitored using the pulse-indicated continuous cardiac output. Renal perfusion was monitored using renal contrast-enhanced ultrasound. CO, cardiac output; CVP, central venous pressure; MAP, mean arterial pressure; PI, peak sonographic signal intensity; RBF, regional blood flow; TTP, time to peak. *Compared with the usual care group, *p* < .05. The differences have been adjusted for baseline value, age, and acute physiology and chronic health evaluation II score.

MAP, central venous pressure (CVP), and cardiac output (CO) were not significantly different between the terlipressin and usual care groups at 24 h after enrollment (Figure 2). The peak sonographic signal intensity at 24 h after enrollment in the terlipressin group (60.5 ± 8.6 dB) was significantly greater than that in the usual care group (52.4 ± 7.0 dB) after adjusting for baseline peak sonographic signal intensity, age, and APACHE II score (mean difference, 7.1 dB; 95% CI, 0.4–13.9; adjusted *p* = .04) (Table 2 and Figure 2).

**Table 2.** Primary and secondary outcomes.

Outcomes^a^	Terlipressin (*N* = 10)	Usual care (*N* = 12)	Unadjusted		Adjustedb		
			Mean difference or odds ratio (95%CI)		*p* value	Mean difference or odds ratio (95%CI)	*p* value
Primary outcome							
PI, dB, mean ± SD	60.5 ± 8.6	52.4 ± 7.0	8.1 (1.1, 15.0)	.025		7.1 (0.4, 13.9)	.04
Secondary outcomes							
TTP, s, mean ± SD	16.0 ± 4.4	21.6 ± 8.3	−5.5 (–11.7, 0.6)	.074		–6.7 (–12.2, −1.2)	.019
RBF, au, mean ± SD	80.9 ± 12.8	70.4 ± 8.6	10.5 (0.9, 20.0)	.034		8.3 (–1.3, 17.9)	.085
MTT, s, mean ± SD	64.1 ± 22.7	73.1 ± 31.4	–9.0 (–33.8, 15.9)	.459		–10.1 (–33.3, 13.1)	.372
RRI, mean ± SD	0.70 ± 0.06	0.71 ± 0.05	0 (–0.03, 0.06)	.651		–0.01 (–0.04, 0.06)	.699
Urine output within 24 hours, mL, mean ± SD	516 ± 305	353 ± 260	163 (–88, 414)	.190		64 (–174, 302)	.579
AKI within 28 days, no. (%)	2 (20)	6 (50)	0.25 (0.04, 1.70)	.157		0.23 (0.02, 2.23)	.203

^a^ PI, TTP, RBF, and MTT were monitored by renal contrast-enhanced ultrasound at 24 h after enrollment. RRI was monitored by renal Doppler ultrasound at 24 h after enrollment.

^b^ PI, TTP, RBF, MTT, and RRI have been adjusted for baseline value, age, and APACHE II score. Urine output within 24 h and AKI within 28 days have been adjusted for age and APACHE II score.

AKI: acute kidney injury; APACHE II: acute physiology and chronic health evaluation II; CI: confidence interval; MTT: mean transit time; PI: peak sonographic signal intensity; RBF: regional blood flow; RRI, renal resistance index; SD: standard deviation; TTP: time to peak.

### Secondary outcomes

After adjusting for baseline value, age, and APACHE II score, the time to peak in the terlipressin group (16.0 ± 4.4s) was significantly lower than that in the usual care group (21.6 ± 8.3s; mean difference, −6.7 s; 95% CI, −12.2–1.2; adjusted *p* = .019). No significant differences in regional blood flow, mean transit time, or renal resistance index at 24 h after enrollment were found. No significant differences in urine output within 24 h and AKI within 28 days were noted between the terlipressin and usual care groups after adjusting for age and APACHE II score (Table 2).

### Exploratory analysis

No significant differences in hemodynamic parameters were noted at baseline between the terlipressin and usual care groups. After 24 h of enrollment, patients in the terlipressin group had a significantly lower heart rate, higher stroke volume, and lower norepinephrine dose than those in the usual care group. No significant differences were found in metabolic data at 24 h after enrollment between the two groups (Table 3).

**Table 3.** Effects of terlipressin on hemodynamic and metabolic data.

	Baseline		24 h after enrollment	
Variables	Terlipressin	Usual care	Terlipressin	Usual care
HR, bpm, mean ± SD	88.3 ± 15.8	84.5 ± 26.3	73.0 ± 11.8^b^*	92.1 ± 16.4
MAP, mmHg, mean ± SD	91.4 ± 12.5	89.0 ± 9.8	82.7 ± 13.5*	91.3 ± 16.4
CVP, mmHg, mean ± SD	10.2 ± 5.0	8.4 ± 2.2	8.7 ± 4.9	7.8 ± 2.2
SV, mL, mean ± SD	74.1 ± 24.1	69.9 ± 27.9	76.8 ± 16.8^b^	57.3 ± 13.5
CO, L/min, mean ± SDa	6.3 ± 1.7	6.0 ± 2.2	5.6 ± 1.4	6.7 ± 2.4
SVRI, dyn.s.cm^–5^.m^2^, mean ± SD	1866 ± 651	2171 ± 921	1888 ± 635	1492 ± 1020
Norepinephrine dose, μg/min, median (IQR)	20 (20, 24)	17 (15, 21)	0 (0, 0)^b^*	5 (0, 17)*
Lactate, mmol/L, median (IQR)	2.1 (1.3, 2.9)	1.5 (1.2, 1.9)	1.9 (1.1, 2.3)	2.1 (1.4, 3.8)*
PH value, mean ± SD	7.42 ± 0.03^b^	7.38 ± 0.05	7.44 ± 0.06	7.42 ± 0.06
SaO_2_, %, mean ± SD	95.8 ± 3.9	96.5 ± 3.4	96.6 ± 1.9	96.5 ± 3.2
ScvO_2_, %, mean ± SD	79.4 ± 4.7	76.0 ± 10.3	76.6 ± 5.8	79.3 ± 5.9
Hemoglobin, g/L, mean ± SD	8.5 ± 1.9	9.9 ± 2.3	8.7 ± 1.3	10.1 ± 2.3

^a^ CO was monitored using the pulse-indicated continuous cardiac output.

^b^ Compared with usual care group, *p* < .05.

*Compared with baseline, *p* < .05. CO: cardiac output; CVP: central venous pressure; HR: Heart rate; IQR: interquartile range; MAP: mean arterial pressure; SaO_2_: oxygen saturation; ScvO_2_: central venous oxygen saturation; SD: standard deviation; SV: stroke volume; SVRI: systemic vascular resistance index.

**Figure 3.**
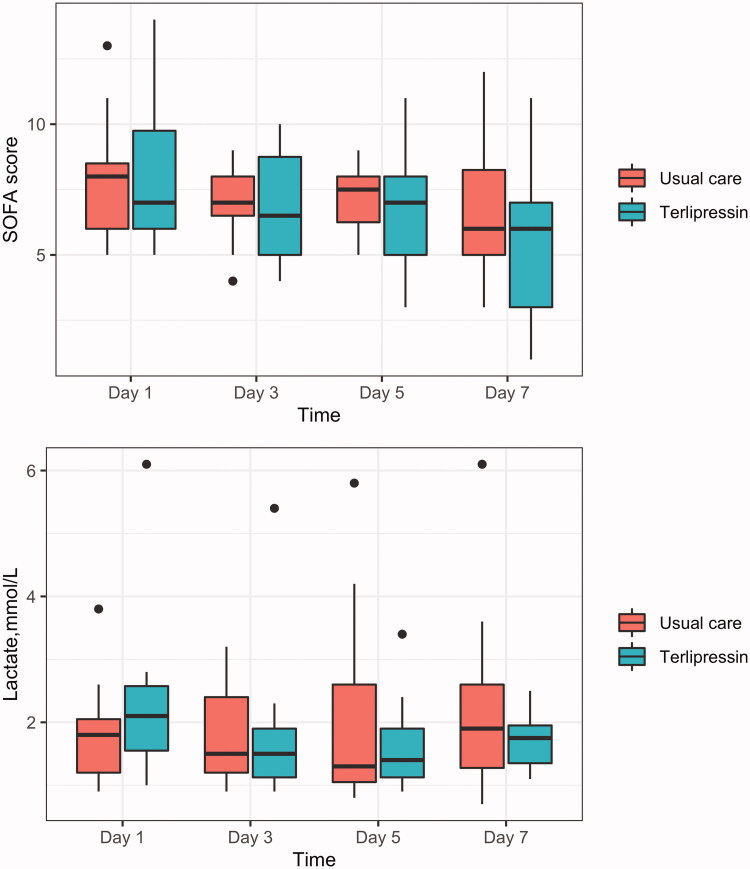
Effects of terlipressin on SOFA score (A) and lactate level (B) in patients with septic shock. Boxplot indicates median (interquartile range) with maximum and minimum values. SOFA, sequential organ failure assessment.

No significant differences were found in the effects of terlipressin versus usual care on organ function. From day 1 to day 7 after enrollment, no significant differences in SOFA scores and lactate levels were noted between the two groups (Figure 3). No statistically significant differences in liver and renal function parameters were noted between the two groups from baseline to 48 h after enrollment (Supplemental Table 1).

The incidence of AKI within 28 days in the terlipressin group (20%) was lower than that in the usual care group (50%), but the difference was not statistically significant (Supplemental Figure 2).

### Adverse events

No statistically significant differences in the incidence of arrhythmology, digital ischemia, diarrhea, or hyponatremia during the ICU stay were found between the terlipressin and usual care groups (Table 4).

**Table 4.** Adverse events.

Events, no. (%)	Terlipressin (*N* = 10)	Usual care (*N* = 12)	*p* value
Arrhythmology	2 (20)	1 (8.3)	.571
Digital ischemia	0 (0)	1 (8.3)	Invalid
Diarrhea	1 (10)	3 (25)	.594
Hyponatremia	2 (20)	3 (25)	>.999
Overall	5 (50)	8 (66.7)	.666

## Discussion

This pilot study aimed to investigate the effects of terlipressin on renal perfusion in patients with septic shock. The results showed that terlipressin added to usual care improved renal perfusion at 24 h after enrollment. This study also demonstrated that terlipressin increased stroke volume, decreased heart rate and norepinephrine dose. Together, the highlight of our study is that terlipressin improves renal perfusion and is a promising vasoactive drug for renal function protective and septic shock prognosis improvement.

Renal contrast-enhanced ultrasound can be used for noninvasive evaluation of renal perfusion in patients with septic shock with good reproducibility. Studies in healthy volunteers have reported that renal contrast-enhanced ultrasound is reproducible [17]. Many factors in the ICU environment can contribute to the heterogeneity of measurements: obesity, passive posture, respiratory movements, and different phases of AKI. However, changes in renal cortical perfusion in septic shock were easily detected despite the heterogeneity caused by these factors [11,18].

Several parameters of renal contrast-enhanced ultrasound can reflect renal perfusion in patients with septic shock. Peak sonographic signal intensity reflects renal perfusion through the measurement of the maximum echo intensity of the contrast agent. Time to peak deals with the time needed to reach the peak sonographic signal intensity after the contrast agent signal appears in the kidney and is associated with renal microvascular dysfunction in septic shock [19]. Regional blood flow depends on the amount of contrast agent in circulation, the patient’s sebum thickness, and renal perfusion, which often lead to large variations in regional blood flow. Mean transit time is the time needed to enhance the renal cortex after contrast agent injection. In this study, we observed an increase in peak sonographic signal intensity and a decrease in time to peak in the terlipressin group compared to the usual care group. The lack of significant differences in regional blood flow and mean transit time between the two groups may be related to the small sample size and large variations.

The therapeutic value of terlipressin and vasopressin in septic shock remains controversial. Previous studies suggested that the therapeutic effects of terlipressin and vasopressin on septic shock included lowering heart rate [20,21], reducing norepinephrine dose [22–24], reducing mechanical ventilation time [25,26], increasing urine output [27], protecting microcirculation and improving vascular reactivity [28], protecting renal function [29–31], promoting organ function recovery [30], and reducing mortality [22]. However, the multicenter randomized controlled trial did not observe a reduction in 28-day mortality, SOFA score, or the number of days alive and free of vasopressors in patients with septic shock treated with terlipressin compared with norepinephrine. The incidence of serious adverse effects was greater in the terlipressin group compared with the norepinephrine group [32]. However, the study was terminated at 50% enrollment, making it underpowered to assess differences in outcomes. This study showed the protective effects of terlipressin on patients with septic shock from the perspective of renal perfusion without an increase in adverse effects.

Results of the CONFIRM trial showed that terlipressin was effective in improving renal function in patients with Type 1 Hepatorenal Syndrome, but was associated with serious adverse events, including respiratory failure [33]. The increased incidence of respiratory failure is possibly related to the cardiovascular and pulmonary effects of terlipressin in patients with Hepatorenal Syndrome [34,35]. Our study and the previous multicenter study [32] did not observe that terlipressin increased the risk of respiratory failure in patients with septic shock, suggesting that the effect of terlipressin on respiratory function in patients with septic shock may be negligible.

We observed a decrease in heart rate, an increase in stroke volume, and a decrease in norepinephrine dose at 24 h after enrollment in the terlipressin group compared with the usual care group. Previous studies supported these results [21,22]. However, it remains unclear whether terlipressin lowers heart rate or norepinephrine raises it. A reduced heart rate could prolong ventricular diastole and increase the volume of returned blood, thereby increasing stroke volume. In addition, an increase in the volume of returned blood could reduce the mean systemic filling pressure and help interstitial fluid flow into the vessels, thereby reducing tissue edema. These effects on macrocirculation suggested that terlipressin might be more effective in patients with septic shock and tachycardia.

Notably, the MAP in the terlipressin group decreased compared with those in the usual care group after 24 h of treatment. The decrease in MAP may be related to the prolonged diastolic period and decreased diastolic pressure. But the effect of the decrease in MAP on extrarenal perfusion was negligible. First, the MAP in the terlipressin group was greater than 65mmhg, which is usually sufficient for tissue perfusion. Second, no significant differences were observed in lactate, SOFA score, and liver function between the two groups, suggesting that the relatively low MAP in the terlipressin group did not lead to extrarenal hypoperfusion.

This study has some strengths. First, we used a prospective randomized controlled method to eliminate the influence of confounders on outcomes. Second, specialized ultrasound physicians who were blinded to the treatment scheme performed the renal contrast-enhanced ultrasound to ensure the accuracy of the measurements. Third, we monitored MAP and cardiac output using the pulse indicator continuous cardiac output to adjust the norepinephrine dose promptly.

The limitations of this study are as follows. First, this was a single-center study with a small sample size, which may limit the generality of the findings. Second, we excluded patients with norepinephrine doses less than 15 μg/min. Because the study used a fixed dose of terlipressin, which may lead to high blood pressure in these patients. Therefore, the findings may not apply to patients with norepinephrine doses less than 15 μg/min. Third, this study did not use a blind design, which might lead to selection bias and measurement bias. To reduce the risk of bias, we used the envelope method to conceal random results to avoid selection bias, and sonographers were blinded to the grouping scheme to reduce measurement bias. Fourth, we assessed the effect of a fixed dose of terlipressin on renal perfusion in patients with septic shock after 24 h of treatment but did not assess whether the effect of terlipressin on renal perfusion was dose-dependent or time-dependent.

Terlipressin added to usual care improves renal perfusion, increases stroke volume, and decreases norepinephrine dose and heart rate in patients with septic shock. Large randomized controlled trials are needed to test the effects of terlipressin on the outcomes of patients with septic shock and to identify which subtypes of septic shock are more likely to benefit from terlipressin treatment.

## Supplementary Material

Supplemental Material

## Data Availability

The datasets used and analyzed during the current study are available from the corresponding author on reasonable request.
