# Supplementary material for: Addition of terlipressin to norepinephrine in septic shock and effect of renal perfusion: a pilot study
Source: Ren Fail. 2022 Jul 20;44(1):1207–15. doi: 10.1080/0886022X.2022.2095286 (PMC9307113; doi:10.1080/0886022X.2022.2095286)

Supplementary material

Supplemental Appendix 1. Method of renal contrast-enhanced ultrasound.

Supplemental Appendix 2. Sample size calculation.

Supplemental Table 1. Effects of terlipressin on liver and kidney function.

Supplemental video 1. Video of renal contrast-enhanced ultrasound.

Supplemental Figure 1. Analysis of renal contrast-enhanced ultrasound.

Supplemental Figure 2. Kaplan–Meier curves for acute kidney injury within 28 days.

Supplemental Appendix 1. Method of renal contrast-enhanced ultrasound

The ultrasound physicians performed renal contrast-enhanced ultrasound using Esaote MyLab Twice ultrasound with CA541 convex probe and SonoVue (Bracco, Italy) as the contrast agent. Renal contrast-enhanced ultrasound was performed as follows. Firstly, we dissolved a Sonovue (59mg) in 5mL of normal saline. The dose of Sonovue used for a single renal contrast-enhanced ultrasound was 0.02 mL/kg. Secondly, we used two-dimensional ultrasound to show the long axis of the kidney. Machine parameters were set as mechanical index 0.07, frequency 10Hz, grayscale 90%, and focusing depth 5cm. Thirdly, an assistant injected prepared Sonovue through a central venous catheter, and renal contrast-enhanced ultrasound was initiated. Renal contrast-enhanced ultrasound images were recorded continuously for 120 seconds (Supplemental video 1).

After the images were collected, we imported the images into the QONTRAST software (Esaote, Italy) for analysis. Three regions of interest in the renal cortex were collected for each image and the regions of interest were 25 square millimeters in size. Movement compensation was applied automatically. A time-intensity curve was generated and renal perfusion parameters were calculated based on this curve. The final analysis results of each patient were averaged and used for statistical analysis.

Supplemental Appendix 2. Sample size calculation

We conducted a preliminary study with a total of 4 patients to calculate the sample size, including 2 patients in the terlipressin group and 2 patients in the usual care group. This is a parallel randomized controlled trial. Enrolled patients were randomly assigned to the terlipressin group and the usual care group in a 1:1 ratio. The primary outcome was peak sonographic signal intensity (a renal perfusion parameter monitored by renal contrast-enhanced ultrasound) at 24 hours after enrollment. According to the results of the preliminary study, the difference in peak sonographic signal intensity at 24 hours after enrollment was 4.2dB between the two groups. The standard deviation was 3.1dB. We set α=0.05, power = 0.8, and the minimum sample size in each group was 10 patients.

Supplemental Table 1. Effects of terlipressin on liver and kidney function

Variables	Groups	Baseline	24 hours after enrollment	48 hours after enrollment
AST, U/L, median (IQR)	Terlipressin	58.0 (30.0,88.0)	38.5 (28.2,80.2)	51.0 (26.2,69.2)
	Usual care	19.0 (15.5,68.0)	25.0 (13.5,55.5)	35.0 (17.5,65.0)
ALT, U/L, median (IQR)	Terlipressin	36.5 (14.8,92.2)	37.5 (17.2,66.0)	29.5 (20.0,58.2)
	Usual care	20.0 (7.5,79.5)	16.0 (9.5,94.0)	22.0 (14.5,54.5)
LDH, U/L, median (IQR)	Terlipressin	279 (230,392)	276 (222,420)	300 (232,391)
	Usual care	271 (196,455)	276 (212,413)	292 (179,426)
Bilirubin, µmol/L, median (IQR)	Terlipressin	25.7 (15.8,50.9)	22.2 (11.3,77.8)	25.6 (11.9,68.2)
	Usual care	19.1 (9.1,27.3)	10.7 (6.2,28.4)	16.9 (7.2,28.0)
Albumin, g/L, median (IQR)	Terlipressin	30.4 (28.3,33.7)	32.5 (29.1,35.0)	31.9 (30.2,34.5)
	Usual care	33.3 (31.6,33.5)	33.7 (29.8,34.5)	32.6 (30.1,33.9)
Serum creatinine, µmol/L, median (IQR)	Terlipressin	71.0 (63.0,95.8)	73.0 (68.2,83.2)	75.0 (65.2,97.2)
	Usual care	82.0 (54.0,106.0)	70.0 (48.5,98.5)	65.0 (50.0,99.0)
BUN, mmol/L, median (IQR)	Terlipressin	8.6 (7.0,11.7)	9.4 (7.9,11.7)	10.6 (8.0,15.9)*
	Usual care	9.2 (4.8,11.2)	8.3 (6.2,10.9)	9.9 (7.7,12.2)

*Compared with baseline, $P<0.05$. ALT, alanine aminotransferase; AST, aspartate aminotransferase; BUN, blood urea nitrogen; IQR, interquartile range; LDH, lactate dehydrogenase.

Supplemental video 1. Video of renal contrast-enhanced ultrasound

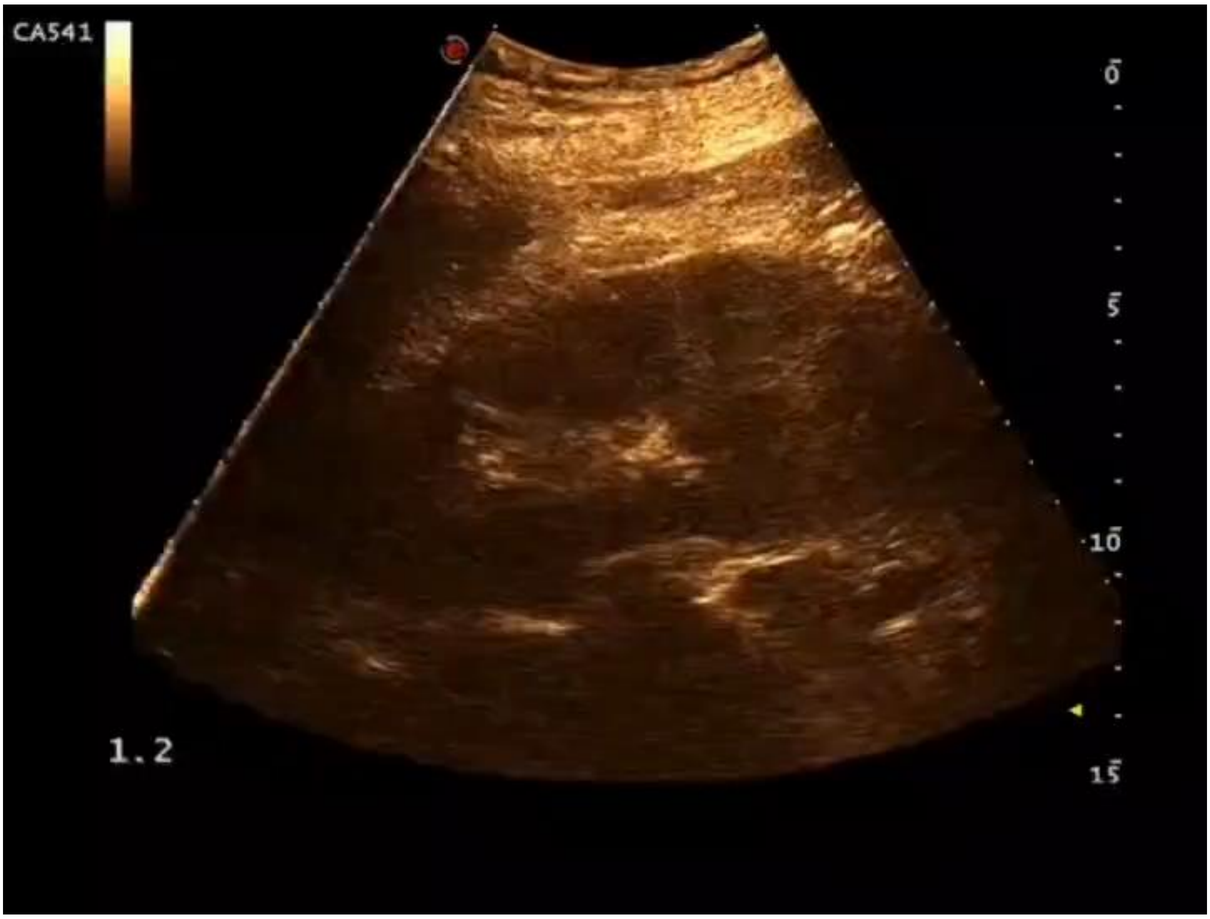

Supplemental Figure 1. Analysis of renal contrast-enhanced ultrasound

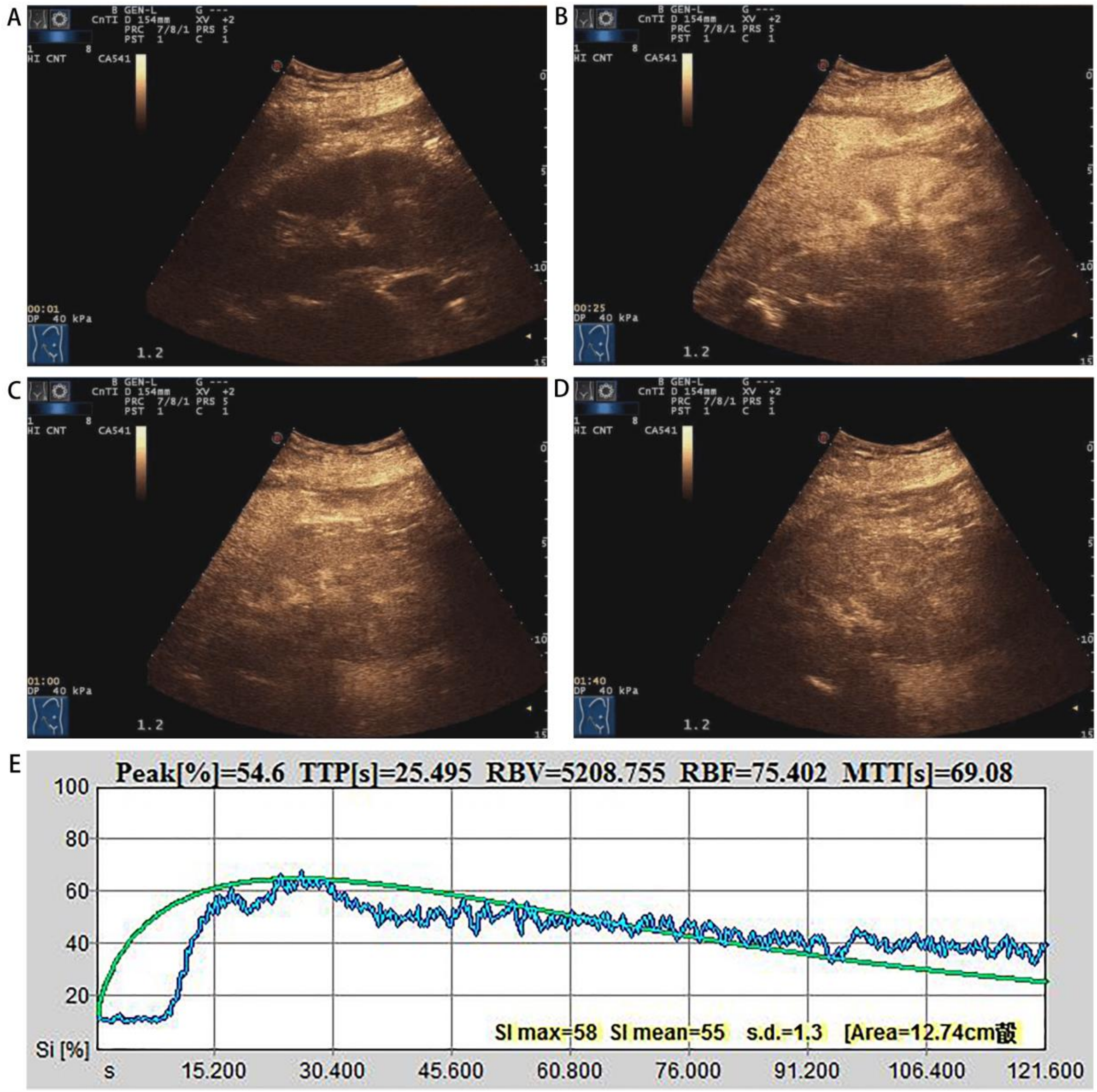

A shows the contrast-enhanced ultrasound image at 1 second; B shows the image at 25 seconds; C shows the image at 60 seconds; D shows the image at 100 seconds. E shows the analysis results of renal contrast-enhanced ultrasound.

Supplemental Figure 2. Kaplan–Meier curves for acute kidney injury within 28 days

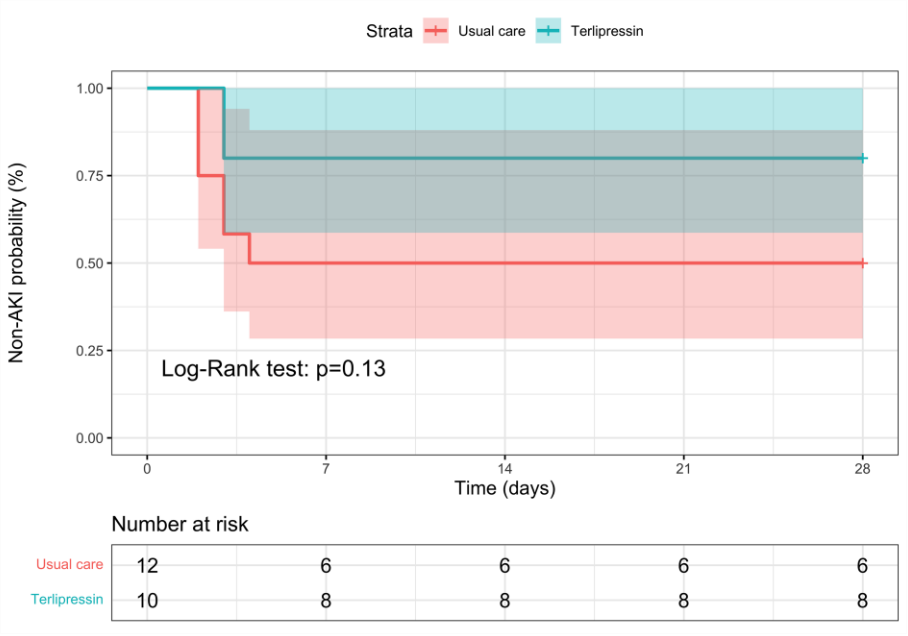

Supplement: Supplemental Material [file IRNF_A_2095286_SM9586.pdf]
